# Perceived Appreciation for Care Associates with Higher Quality Caregiving Day-to-Day

**DOI:** 10.1093/geroni/igab046.2925

**Published:** 2021-12-17

**Authors:** Kylie Meyer, Arielle Chinea, Sara Masoud, Kevin Hamilton, Ashlie Glassner, Jing Wang, Carole White

**Affiliations:** 1 UT Health Sciences at San Antonio, San Antonio, Texas, United States; 2 UT Health San Antonio, San Antonio, Texas, United States; 3 University of Texas Health Science Center at San Antonio, San Antonio, Texas, United States

## Abstract

Family members are critical to dementia care and the U.S. long-term services system. Yet, little is known about how to support the quality of care provided by family members, who often receive little training. We hypothesize that on days when caregivers feel more appreciated, they report providing a higher quality of care. To test this hypothesis, we asked spousal dementia caregivers (N=21) to complete 14 daily surveys that asked about their daily caregiving experiences. Our measure for “quality of care” was based on the Exemplary Caregiving Scale, and included 3-items pertaining to provision of care (e.g., “You considered your spouse's wishes and opinions when providing assistance”). Response options included “Most of the time,” “Some of the time,” and “Never”; scores were summed (range 0 to 6). Caregivers were also asked to what extent their spouse appreciated the care provided (“Not at all,” “Some,” or “A lot”). We applied multi-level mixed models to the data, and controlled for age, gender, Hispanic ethnicity, number of behavioral symptoms of dementia each day and months since diagnosis. In adjusted models, we found that on days when caregivers believed care recipients appreciated care provided “Some” or “A lot,” they reported providing higher quality care (B=0.52, p=0.010 and B=0.79, p<0.001, respectively) compared with days when caregivers believed care recipients appreciated care provided “Not at all”. Preliminary results may inform programs to support caregivers’ ability to provide high quality care (e.g., by helping caregivers to perceive rewards) and to identify caregivers at risk of providing low-quality care.

